# The novel fibrosis index at diagnosis may predict all-cause mortality in patients with antineutrophil cytoplasmic antibody-associated vasculitis without substantial liver diseases

**DOI:** 10.6061/clinics/2021/e2501

**Published:** 2021-03-30

**Authors:** Jung Yoon Pyo, Sung Soo Ahn, Lucy Eunju Lee, Gwang-mu Choi, Jason Jungsik Song, Yong-Beom Park, Sang-Won Lee

**Affiliations:** I Division of Rheumatology, Department of Internal Medicine, Yonsei University College of Medicine, Seoul, Republic of Korea; II Department of Medicine, Yonsei University College of Medicine, Seoul, Republic of Korea; III Institute for Immunology and Immunological Diseases, Yonsei University College of Medicine, Seoul, Republic of Korea

**Keywords:** Antineutrophil Cytoplasmic Antibody-Associated Vasculitis, Novel Fibrosis Index, Predict, All-Cause Mortality

## Abstract

**OBJECTIVES:**

Antineutrophil cyto plasmic antibody-associated vasculitis (AAV) is a fatal disease. Currently, predictors of mortality due to AAV are based on the distribution of organ involvement. The novel fibrosis index (NFI) is an index composed of laboratory results that reflect the degree of liver fibrosis. This study aimed to evaluate whether NFI can predict poor outcomes in patients with AAV without substantial liver disease.

**METHODS:**

A total of 210 patients with immunosuppressive drug-naïve AAV were retrospectively reviewed. NFI was calculated as follows: NFI=(serum bilirubin × (alkaline phosphatase)^2^)/(platelet count×(serum albumin)^2^). NFI cut-off was set at 1.24 (the highest quartile). Poor outcomes were defined as all-cause mortality, relapse, and end-stage renal disease (ESRD).

**RESULTS:**

During the median 34.5 months of follow-up, 21 patients (10%) died, 72 patients (34.3%) relapsed, and 38 patients (18.1%) had ESRD due to AAV progression. The median calculated NFI was 0.61, and it was higher in AAV patients with all-cause mortality than in those without mortality, but the difference was not statistically significant (1.26 *vs*. 0.59). AAV patients with NFI at diagnosis ≥1.24 exhibited a significantly lower cumulative patient survival rate than those with NFI at diagnosis <1.24 (*p*=0.002). Multivariate Cox hazard model analysis showed that NFI at diagnosis ≥1.24 was an independent predictor of all-cause mortality in AAV (hazard ratios [HR] 2.850, 95% confidence interval [CI] 1.026, 7.910).

**CONCLUSIONS:**

NFI ≥1.24, which may be an independent predictive marker for all-cause mortality in AAV patients without substantial liver disease.

## INTRODUCTION

Antineutrophil cytoplasmic antibody (ANCA)-associated vasculitis (AAV) is a systemic vasculitis characterized by necrotizing vasculitis in small-sized vessels, arterioles, venules, capillaries, and, occasionally, arteries. AAV has three subtypes based on histological and clinical features: microscopic polyangiitis (MPA), granulomatosis with polyangiitis (GPA), and eosinophilic granulomatosis with polyangiitis (EGPA) (1). Various classification criteria and definitions have been suggested, including the 2007 European Medicine Agencies algorithm for AAV and the 2012 revised International Chapel Hill Consensus Conference (CHCC) Nomenclature of Vasculitides, widely used to diagnose AAV (1,2). The disease activity of AAV is assessed and expressed by the Birmingham vasculitis activity score (BVAS), which consists of nine items (3). In addition, the vasculitis damage index (VDI) is used to assess the irreversible damage caused by AAV, and a five-factor score (FFS) is used to predict prognosis (4,5).

BVAS or VDI include several major organs, but they do not contain the symptoms or damages of liver involvement in AAV. Similarly, a previous study reported that the progression of liver involvement or autoimmune hepatitis was rarely observed in patients with AAV (6). Meanwhile, another earlier study reported that liver fibrosis indices were associated with all-cause mortality in patients with AAV without substantial chronic liver disease (7). Concerning AAV, a hypothesis is more persuasive that these results might have been related to the variables’ association of the variables. These variables comprise the liver fibrosis indices formulas, with the cross-sectional inflammatory burden and its related poor outcomes of AAV, rather than the direct association between the liver fibrosis indices and liver involvement of AAV.

Recently, two similar liver fibrosis indices have been introduced: fibrosis cirrhosis index (FCI) and novel fibrosis index (NFI). Both FCI and NFI are newly designed indicators to predict the degree of liver fibrosis non-invasively. FCI is calculated by a formula consisting of four parameters: FCI=(serum bilirubin×alkaline phosphatase)/(platelet count×serum albumin) (8). NFI is a modified formula by squaring both alkaline phosphatase in the molecule of FCI and serum albumin in the denominator of the formula FCI: NFI=(serum bilirubin×(alkaline phosphatase)^2^)/(platelet count×(serum albumin)^2^) (9).

NFI is a modified formula by squaring both alkaline phosphatase in the FCI molecule and serum albumin in the FCI formula denominator. NFI amplifies the reflected contribution of alkaline phosphatase, which shows a positive correlation with liver fibrosis, and serum albumin, which shows a negative correlation (9). Among the NFI variables, few previous reports on the association of AAV prognosis with bilirubin or alkaline phosphatase at diagnosis. Still, the association of AAV prognosis and either platelet count or serum albumin at diagnosis has already been demonstrated (10,11). Therefore, it can be speculated that NFI at diagnosis could predict poor outcomes of AAV during follow-up. However, there has been no report on the clinical significance of NFI at diagnosis in the disease course of AAV. Hence, in this study, we investigated whether NFI at diagnosis might be associated with AVV’s cross-sectional activity and could predict the poor outcomes of AAV, particularly all-cause mortality, during follow-up in immunosuppressive drug-naïve patients with AAV without substantial chronic liver disease.

## MATERIALS AND METHODS

### Patients

We retrospectively reviewed the medical records of 210 immunosuppressive drug-naïve AAV patients, who had been classified as AAV based on the 2007 EMA algorithm and the 2012 CHCC definitions at the Division of Rheumatology, Department of Internal Medicine, Yonsei University College of Medicine, Severance Hospital between October 2000 and March 2020 (1,2). BVAS and FFS were collected, and if not available, they were calculated based on clinical and laboratory data documented in the medical records. Confirmation of ANCA, both by an indirect immunofluorescence assay (IFA) for perinuclear (P)-ANCA and cytoplasmic (C)-ANCA and antigen-specific assays for myeloperoxidase (MPO)-ANCA and proteinase 3 (PR3)-ANCA were also included in the medical records. Patients negative by antigen-specific assay but positive for ANCA by IFA were considered to have MPO-ANCA or PR3-ANCA when AAV was strongly suspected per clinical and laboratory features (12). All patients included in this study had been followed up for at least three months or longer. Patients had no serious medical conditions, such as coexisting malignancies, serious infections, or chronic liver diseases affecting the NFI value at diagnosis. In addition, they had never received immunosuppressive drugs before the diagnosis. This study was approved by the Institutional Review Board of Severance Hospital (4-2017-0673). The need for patients’ written informed consent was waived, as this was a retrospective study.

### Exclusion of substantial liver diseases

Blood tests identified substantial liver diseases, liver function, viral hepatitis A, B, and C, and imaging studies, such as computed tomography or ultrasonography at diagnosis. In addition, they were confirmed by the 10^th^ revised International Classification of Diseases.

### Clinical data at diagnosis and during follow-up

At diagnosis, age, sex, body mass index (BMI), and smoking history were obtained. AAV subtypes, ANCA positivity, BVAS, and FFS were also collected as AAV-specific indices. We assessed for chronic kidney disease (stages 3-5), diabetes mellitus, hypertension, and interstitial pneumonia. Erythrocyte sedimentation rate (ESR) and C-reactive protein (CRP) levels were collected as acute-phase reactants, platelet count, prothrombin time, serum albumin, alkaline phosphatase, aspartate aminotransferase, alanine aminotransferase, and total bilirubin were also collected as liver-related variables. We also reviewed medications administered during follow-up, including glucocorticoid, cyclophosphamide, rituximab, azathioprine, mycophenolate mofetil, tacrolimus, and methotrexate.

### Poor outcomes

All-cause mortality, relapse, and end-stage renal disease (ESRD) were evaluated as poor AAV outcomes during follow-up. The follow-up period was defined as the duration between the diagnosis and the last visit for surviving patients. Deceased patients were defined as the duration between diagnosis and death. Patients who had relapse and ESRD were defined as the duration from diagnosis until relapse or the renal replacement therapy initiation, respectively.

### Equation for FCI and NFI

FCI and NFI were calculated using the following formulas: FCI=(serum bilirubin×alkaline phosphatase)/(platelet count×serum albumin) (8), and NFI=(serum bilirubin×(alkaline phosphatase)^2^)/(platelet count×(serum albumin)^2^) (9).

### Statistical analyses

All statistical analyses were conducted using the SPSS software (version 23 for Windows; IBM Corp., Armonk, NY, USA). Continuous variables were expressed as median (interquartile range, IQR), and categorical variables were expressed as numbers and percentages. Significant differences in categorical variables and continuous variables between the two groups were analyzed using the Chi-square and Fisher’s exact tests and the Mann-Whitney test. The correlation coefficients between the two groups were obtained using Pearson or Spearman correlation analyses. Comparing the cumulative survival rates between the two groups was performed using the Kaplan-Meier survival analysis with the log-rank test. The multivariable Cox hazards model analysis using statistical significance variables in the univariable Cox hazards model analysis was conducted to obtain the hazard ratios (HRs) during the considerable follow-up duration. The relative risk (RR) of A for B was analyzed using contingency tables and the chi-square test. Statistical significance was set at *p*<0.05.

## RESULTS

### Characteristics of AAV patients at diagnosis

At the time of diagnosis, the patients’ median age was 59.0 years, and 32.5% were men. The median BMI was 22.2 kg/m^2^, and only seven patients had ever smoked but were not current smokers. The most common AAV subtype and detected ANCA type were MPA (55.2%) and MPO-ANCA (or P-ANCA) (66.7%). The median BVAS and FFS were 12.0 and 1.0, respectively. The most common comorbidity was hypertension (46.7%), followed by chronic kidney disease (stage 3-5) (28.6%). The median ESR, CRP, NFI, and FCI were 60.5 mm/hr, 13.2 mg/L, 0.61, and 0.032, respectively. The median liver-related variables are presented in Table 1.

During the follow-up period, 21 patients (10%) died, 72 patients (34.3%) relapsed, and 38 patients (18.1%) had ESRD due to AAV progression. The median follow-up duration until each event was 34.5 months, 22.8 months, and 30.5 months, respectively. Glucocorticoids were administered to 91.4% of patients, and both cyclophosphamide (48.1%) and azathioprine (47.6%) were most commonly used as induction therapy and maintenance therapy (Table 1). Cyclophosphamide was administered intravenously in all patients, and maintenance therapy continued for at least two years in most patients.

### Correlation of continuous variables at diagnosis

NFI and FCI tended to be correlated with AAV’s cross-sectional activity based on BVAS but did not reach statistical significance. Furthermore, neither NFI nor FCI was significantly correlated with age, BMI, FFS, ESR, or CRP. In addition, except for liver-related variables associated with NFI and FCI, prothrombin time, aspartate aminotransferase, and alanine aminotransferase were significantly correlated with both NFI and FCI, as expected (Table 2).

### Comparison of FCI and NFI at diagnosis based on each poor outcome

**Figure 1:**
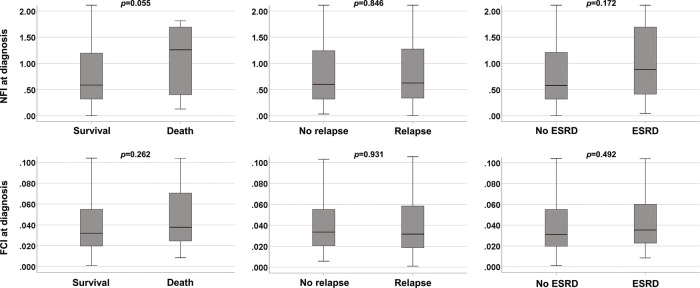
Comparison of FCI and NFI at diagnosis based on each poor outcome. Among six conditions, only NFI at diagnosis in deceased AAV patients tended to be increased compared to survived AAV patients, but it did not reach a statistical significance. NFI: novel fibrosis index; ESRD: end-stage renal disease; FCI: fibrosis cirrhosis index.

Deceased AAV patients exhibited a higher NFI at diagnosis than survived AAV patients, but the difference was not statistically significant (1.26 *vs*. 0.59, *p*=0.055). However, there were no statistically significant NFI differences at diagnosis between AAV patients with relapse and those without relapse, or AAV patients with ESRD and those without ESRD. On the other hand, FCI at diagnosis did not differ between the three groups classified according to all-cause mortality, relapse, or ESRD occurrence (Figure 1).

### Cut-offs of NFI and FCI at diagnosis for each poor outcome

To obtain the cut-off values of NFI and FCI at diagnosis for each poor outcome, we conducted the ROC curve analysis but could not obtain the cut-offs. Instead, we arbitrarily defined the NFI and FCI’s cut-off values as the lower limit of the highest quartile at diagnosis: NFI≥1.24 and FCI≥0.057.

### Comparison of cumulative survival rates

**Figure 2:**
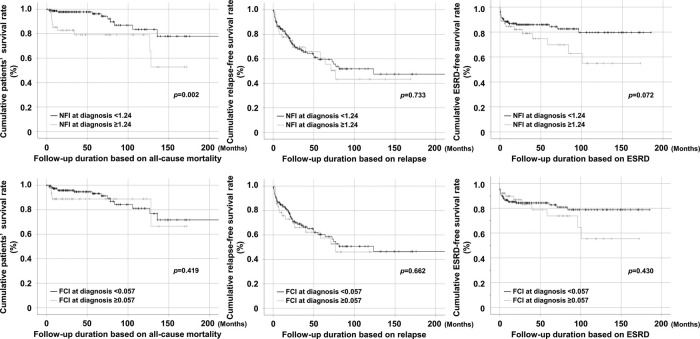
Comparison of the cumulative survival rates. Among six conditions, it was found that only AAV patients with NFI at diagnosis ≥1.24 exhibited a significantly lower cumulative patients’ survival rate than those without. NFI: novel fibrosis index; ESRD: end-stage renal disease; FCI: fibrosis cirrhosis index.

AAV patients with NFI at diagnosis ≥1.24 exhibited a significantly lower cumulative patient survival rate than those with NFI at diagnosis <1.24 (*p*=0.002). However, the cumulative relapse-free and ESRD-free survival rates were not different between AAV patients with NFI at diagnosis ≥1.24 and those without. Meanwhile, FCI at diagnosis ≥0.057 could not predict any AAV’s poor outcome (Figure 2).

### Cox hazards model analysis for all-cause mortality

To clarify whether NFI at diagnosis ≥1.24 might be an independent predictor of all-cause mortality during follow-up, we conducted univariate and multivariate Cox hazards model analysis using variables at diagnosis for all-cause mortality. Liver-related variables were excluded from the Cox analyses to minimize confounding effects on the association between NFI and all-cause mortality. In the univariate analysis, age, male sex, BVAS score, hypertension, interstitial lung disease, CRP, and NFI at diagnosis ≥1.24, were significantly associated with all-cause mortality. In the multivariable analysis, age (HR 1.049, 95% confidence interval [CI] 1.05, 1.095), BVAS (HR 1.082, 95% CI 1.010, 1.159), interstitial lung disease (HR 4.728, 95% CI 1.878, 11.901), and NFI at diagnosis ≥1.24 (HR 2.850, 95% CI 1.026, 7.910) were found to be independent predictors of all-cause mortality during follow-up in AAV patients (Table 3).

### Relative Risk of NFI at diagnosis ≥1.24 for all-cause mortality

**Figure 3:**
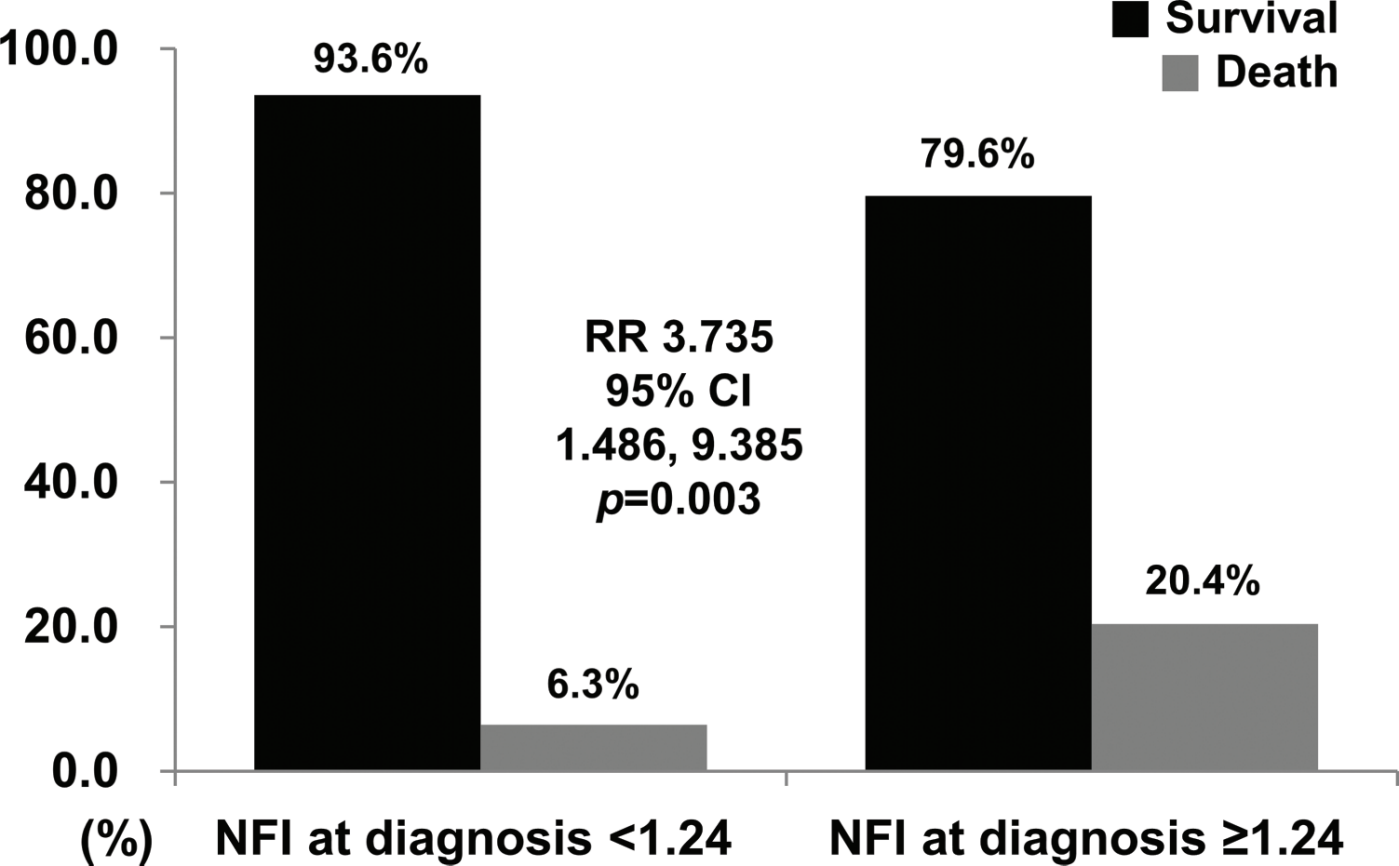
Relative Risk for all-cause mortality. AAV patients with NFI at diagnosis ≥1.24 had a significantly higher risk for all-cause mortality than those with NFI at diagnosis <1.24 (RR 3.735). NFI: novel fibrosis index; RR: relative risk; CI: confidence interval.

When we classified AAV patients into two groups based on the calculated cut-off of NFI, 54 of 210 patients were divided into the group with NFI at diagnosis ≥1.24. All-cause mortality was identified more frequently in AAV patients with NFI at diagnosis ≥1.24 than in those with NFI at diagnosis <1.24 (20.4% *vs*. 6.3%, *p*=0.003). Furthermore, AAV patients with NFI at diagnosis ≥1.24 had a significantly higher risk for all-cause mortality than those with NFI at diagnosis <1.24 (RR 3.735, 95% CI 1.468, 9.385) (Figure 3).

## DISCUSSION

In this study, we investigated whether NFI, one of the indices for liver fibrosis, might be associated with AAV’s cross-sectional activity and could predict poor outcomes, particularly all-cause mortality in immunosuppressive drug-naïve patients with AAV. We found four valuable results as follows: first, NFI could reflect the cross-sectional activity of AAV based on BVAS or acute-phase reactants, including ESR and CRP. Second, when the cut-off of NFI at diagnosis was defined as the lower limit of the higher quartile, AAV patients with NFI at diagnosis ≥1.24 exhibited a significantly lower cumulative patient survival rate than those with NFI at diagnosis <1.24. Third, in the multivariable Cox hazards model analysis, NFI at diagnosis ≥1.24 could independently predict all-cause mortality during follow-up together with age, BVAS, and interstitial lung disease at diagnosis. Lastly, AAV patients with NFI at diagnosis ≥1.24 had a significantly higher risk for all-cause mortality than those with NFI at diagnosis <1.24 (RR 3.735).

When we assessed the frequency of affected organs based on the nine items of the BVAS in our study population, the most commonly affected area was renal manifestation (60.0%), followed by pulmonary (57.1%), and ear nose throat (ENT) (44.8%). We wondered which specific organ involvement was associated with NFI and found that only the ENT manifestation frequency among the nine manifestations exhibited a significant difference in NFI. Ninety-four AAV patients with ENT manifestation exhibited a statistically significantly higher median NFI than 116 AAV patients without ENT manifestation (0.74 *vs*. 0.50, *p*=0.024). Although it has been controversial whether sinusitis might be a typical surrogate marker for GPA, sinusitis is the most common symptom of ENT involvement in AAV (13). We conducted a literature review on the association of sinusitis with the four variables that compose an NFI formula and found a previous study reported that serum albumin was significantly lower in patients with chronic rhinosinusitis than those without (14). However, in this study, serum albumin levels did not differ between AAV patients with and without ENT manifestations. In addition, since serum albumin is more clinically linked to renal manifestation. It is difficult to explain the difference in NFI between AAV patients with and those without ENT manifestation by the results of this previous study alone.

In this study, when the ROC curve dependent variable was designated as all-cause mortality or ESRD, statistical significance was not obtained. There are cases where there is no or low statistical significance when calculating the cut-off using the ROC curve because the follow-up duration based on all-cause mortality or ESRD occurrence was not considered. Conversely, the Kaplan-Meier survival analysis used to evaluate the predictive indicator potential requires follow-up duration based on each poor prognosis. On the other hand, in the case of failure to obtain the cut-off using the ROC curve, the highest or the lowest tertile or quartile is occasionally used to obtain a statistically significant cut-off. We also designated the NFI and FCI’s highest quartiles as cut-off values for all-cause mortality and ESRD occurrence in this study. They exhibited statistical significance or a trend of significance in the Kaplan-Meier analysis.

**Supplementary Figure 1:**
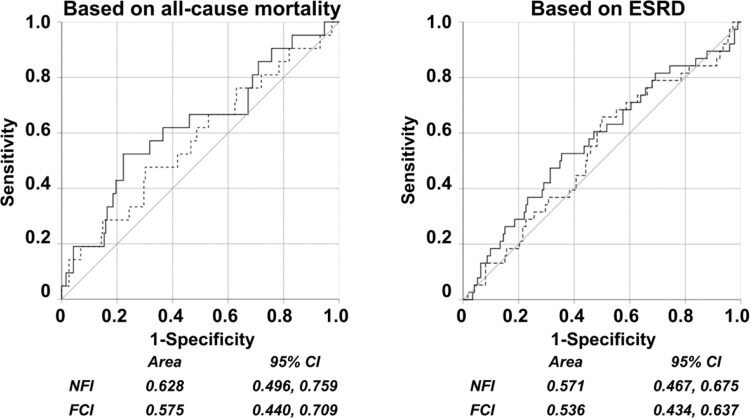
Comparison of the areas under the curve between NFI and FCI in the ROC curve analysis based on all-cause mortality and ESRD occurrence. NFI: novel fibrosis index; FCI: fibrosis cirrhosis index; ROC: Receiver Operating Characteristic; ESRD: endstage renal disease.

Despite no statistical significance, NFI and FCI areas at diagnosis for all-cause mortality and ESRD using the ROC curve were qualitatively compared. First, in the ROC curve analysis based on all-cause mortality, NFI at diagnosis showed a wider area under the curve than FCI at diagnosis. Moreover, in the ROC curve analysis based on ESRD, NFI at diagnosis also showed a wider area under the curve than FCI at diagnosis (Appendix - Supplementary Figure 1). Therefore, although the statistical significance was low and the follow-up period was not taken into account, NFI at diagnosis tended to better predict the occurrence of all-cause mortality and ESRD during follow-up than FCI at diagnosis.

The exact mechanism of how NFI at diagnosis independently predicts all-cause mortality during AAV patients’ follow-up is unclear. However, we made some assumptions. Hypoalbuminemia is a well-known major risk factor for poor prognosis, including all-cause mortality and ESRD occurrence in AAV patients (15). Since serum albumin is in the NFI formula’s denominator, NFI and serum albumin theoretically show an inverse correlation. Therefore, it can be inferred that the smaller the albumin level, the higher the NFI. The highest NFI quartile at diagnosis indicates a lower level of serum albumin could predict all-cause mortality in AAV patients. The Pearson correlation analysis performed in our study did not reach statistical significance but showed a tendency toward a negative correlation (r=-0.119, *p*=0.084). Using the Spearman correlation analysis, serum albumin levels were significantly and inversely correlated with NFI (rho=-0.322, *p*<0.001). These results support our assumption that NFI can predict all-cause mortality through serum albumin levels.

In terms of platelet count, there have been studies demonstrating that platelet counts reflect AAV’s inflammatory burden, correlate with BVAS, and may predict poor outcomes in AAV patients (10,16). Since platelet count is also in the denominator of NFI’s formula, theoretically, the larger the platelet counts, the smaller the NFI. However, the results of our study disagreed. When we focus on two variables in the denominator, serum albumin can be considered to have a direct effect on NFI’s increase more than platelet count. Therefore, we concluded that the highest quartile of NFI at diagnosis made a more direct contribution to predicting all-cause mortality through reduced serum albumin, which could reflect the high degree of inflammation and malnutrition.

In the Kaplan-Meier survival analysis, the cumulative ESRD-free survival rate in AAV patients with NFI at diagnosis ≥1.24 was lower than that in AAV patients with NFI at diagnosis <1.24 (*p*=0.072). However, the difference was not statistically significant. This result may suggest that analyzing a larger number of patients can yield statistically significant results.

How can NFI at diagnosis, which belongs to the highest quartile, tend to predict ESRD during follow-up in AAV patients? In terms of comorbidity of chronic kidney disease, AAV patients with chronic kidney disease at diagnosis showed higher NFI than those without (0.86 *vs*. 0.55, *p*=0.060). Therefore, NFI can be assumed to reflect the accompanying chronic kidney disease at diagnosis, which could potentially predict ESRD occurrence in AAV patients (17).

In terms of serum albumin, high NFI means reduced serum albumin at diagnosis, as described above. Reduced serum albumin, in turn, is associated with renal manifestation and increased BVAS and is another crucial risk factor for ESRD (3,18). Using the univariable Cox hazards model analysis, serum albumin level at diagnosis was significantly associated with ESRD during the follow-up period based on ESRD occurrence (HR 0.585, 95% CI 0384, 0.890). These results support our assumption that NFI can predict ESRD through serum albumin levels.

NFI was originally a liver fibrosis index (19). Nevertheless, how can it predict ESRD? Although the cells involved in liver fibrosis and renal fibrosis are different, biological factors, such as cytokines and chemokines that ultimately induce and promote collagen deposition in organs, may share common signal pathways in the two different fibrosis processes (9,20). Therefore, although it is difficult to explain the exact mechanism, it is expected that the liver fibrosis index can predict the process of renal fibrosis to some extent.

To the best of our knowledge, this is the first study to demonstrate that NFI at diagnosis could independently predict all-cause mortality in immunosuppressive drug-naïve AAV patients without chronic liver diseases. The discovery of a new predictor of all-cause mortality in AAV patients is an essential advantage of our research. In addition, our study was conducted in a single institution, which may be a drawback. Still, it is also an advantage in that chronic liver diseases at the time of diagnosis and during the follow-up period were strictly excluded from this study.

Our study has several limitations. Although chronic liver diseases were excluded from this study, there was no information on liver fibrosis examined by transient elastography or ultrasonography in high NFI patients. The disadvantage is that there is no serial information on liver fibrosis in deceased AAV patients. Due to a retrospective single institutional study’s limitations, there is a possibility of selection bias, and missing data among clinical data could not be completely excluded. Future prospective longitudinal studies with a larger sample of AAV patients and serial information regarding the liver fibrosis presence measured by transient elastography will validate and support our research and provide dynamic data on NFI in AAV patients.

## CONCLUSION

Our study demonstrated that NFI at diagnosis was not associated with the cross-sectional BVAS. In contrast, the highest quartile of NFI at diagnosis could independently predict all-cause mortality during follow-up in immunosuppressive drug-naïve AAV patients without substantial liver diseases.

## AUTHOR CONTRIBUTIONS

Pyo JY and Lee SW participated in the research design, writing of the manuscript final version and the research’s performance. Pyo JY, Ahn SS, Lee LE and Lee SW contributed to the data acquisition and interpretation. Pyo JY, Ahn SS and Lee SW participated in the manuscript drafting. Choi GM conducted statistical analyses and validated the interpretation. In particular, Lee LE helped with English editing. Song JJ and Park YB reviewed the drafting and final version of the manuscript. All authors read and approved the final version of the manuscript.

## Figures and Tables

**Table 1:** Characteristics of AAV patients at diagnosis and during follow-up (N=210).

AAV patients	Values
**At the time of diagnosis**	
**Demographic data**	
Age (years)	59.0 (20.3)
Male gender (N, (%))	68 (32.4)
Body mass index (kg/m^2^)	22.2 (4.4)
Smoking history (N, (%))	7 (3.3)
**AAV subtypes (N, (%))**	
MPA	116 (55.2)
GPA	52 (24.8)
EGPA	42 (20.0)
**ANCA positivity (N, (%))**	
MPO-ANCA (or P-ANCA) positivity	140 (66.7)
PR3-ANCA (or C-ANCA) positivity	35 (16.7)
Both ANCA positivity	9 (4.3)
ANCA negativity	44 (21.0)
**AAV-specific indices**	
BVAS	12.0 (11.0)
FFS	1.0 (2.0)
**Comorbidities at diagnosis (N, (%))**	
Chronic kidney disease (stage 3-5)	60 (28.6)
Diabetes mellitus	55 (26.2)
Hypertension	98 (46.7)
Interstitial lung disease	55 (26.2)
**Acute-phase proteins**	
ESR (mm/hr)	60.5 (70.3)
CRP (mg/L)	13.2 (70.6)
**Liver-related variables**	
Platelet count (x1,000/mm^3^)	304.5 (158.0)
Prothrombin time (INR)	1.0 (0.2)
Serum albumin (g/dL)	3.6 (1.2)
Alkaline phosphatase (IU/L)	70.5 (37.8)
Aspartate aminotransferase (IU/L)	18.0 (9.0)
Alanine aminotransferase (IU/L)	15.0 (15.0)
Total bilirubin (mg/dL)	0.5 (0.2)
**NFI**	0.61 (0.95)
**FCI**	0.032 (0.037)

During the follow-up period	
**Follow-up duration (months)**	34.5 (64.6)
**Poor outcomes during follow-up (N, (%))**	
All-cause mortality (N, (%))	21 (10.0)
Follow-up duration based on all-cause mortality (months)	34.5 (64.7)
Relapse (N, (%))	72 (34.3)
Follow-up duration based on relapse (months)	22.8 (44.0)
ESRD (N, (%))	38 (18.1)
Follow-up duration based on ESRD (months)	30.5 (69.4)
**Medications administered during follow-up (N, (%))**	
Glucocorticoid	192 (91.4)
Cyclophosphamide	101 (48.1)
Rituximab	31 (14.8)
Azathioprine	100 (47.6)
Mycophenolate mofetil	23 (11.0)
Tacrolimus	11 (5.2)
Methotrexate	19 (0.0)

Values are expressed as median (interquartile range [IQR]) or N (%).

AAV: ANCA-associated vasculitis; ANCA: antineutrophil cytoplasmic antibody; MPA: microscopic polyangiitis; GPA: granulomatosis with polyangiitis; EGPA: eosinophilic GPA; MPO: myeloperoxidase; P: perinuclear; PR3: proteinase 3; C: cytoplasmic; BVAS: Birmingham vasculitis activity score; FFS: five-factor score; ESR: erythrocyte sedimentation rate; CRP: C-reactive protein; NFI: novel fibrosis index; FCI: fibrosis cirrhosis index; ESRD: end-stage renal disease.

**Table 2:** Correlation of continuous variables with either NFI or FCI at diagnosis in AAV patients.

	Based on NFI		Based on FCI	
Variables	Correlation coefficient (r)	*p*-value	Correlation coefficient (r)	*p*-value
Age	0.061	0.380	0.063	0.366
BMI	0.046	0.511	0.046	0.503
BVAS	0.133	0.054	0.133	0.055
FFS	0.046	0.508	0.046	0.504
ESR	0.009	0.898	0.010	0.885
CRP	-0.013	0.846	-0.005	0.937

Liver-related variables*				
Prothrombin time (INR)	0.181	0.009	0.184	0.009
Aspartate aminotransferase (IU/L)	0.765	<0.001	0.770	<0.001
Alanine aminotransferase (IU/L)	0.635	<0.001	0.640	<0.001

NFI: novel fibrosis index; FCI: fibrosis cirrhosis index; AAV: ANCA-associated vasculitis; BMI: body mass index; BVAS: Birmingham vasculitis activity score; FFS: five-factor score; ESR: erythrocyte sedimentation rate; CRP: C-reactive protein.

* Platelet count, serum albumin, alkaline phosphatase, and bilirubin, which are variables of the NFI and FCI equations, were not included in this table.

**Table 3:** Cox hazards model analysis of variables at diagnosis for all-cause mortality during follow-up in AAV patients.

Variables	Univariable			Multivariable		
	HR	95% CI	*p*-value	HR	95% CI	*p*-value
Age	1.060	1.019, 1.104	0.004	1.049	1.005, 1.095	0.029
Body mass index	1.115	0.962, 1.293	0.147			
Male gender	2.410	1.021, 5.690	0.045	2.289	0.899, 5.830	0.082
Smoking history	1.964	0.262, 14.734	0.512			
MPO-ANCA (or P-ANCA) positivity	1.784	0.682, 4.664	0.238			
PR3-ANCA (or C-ANCA) positivity	1.036	0.348, 3.088	0.949			
BVAS	1.081	1.002, 1.143	0.007	1.082	1.010, 1.159	0.024
Chronic kidney disease (stage 3-5)	1.985	0.833, 4.733	0.122			
Diabetes mellitus	1.015	0.393, 2.619	0.976			
Hypertension	3.132	1.145, 8.566	0.026	1.414	0.471, 4.241	0.536
Interstitial lung disease	5.614	2.307, 13.663	<0.001	4.728	1.878, 11.901	0.001
ESR	1.009	0.998, 1.020	0.108			
CRP	1.007	1.001, 1.014	0.022	0.999	0.991, 1.007	0.768
NFI at diagnosis ≥1.24	3.627	1.538, 8.556	0.003	2.850	1.026, 7.910	0.044

AAV: ANCA-associated vasculitis; ANCA: antineutrophil cytoplasmic antibody; MPO: myeloperoxidase; P: perinuclear; PR3: proteinase 3; C: cytoplasmic; BVAS: Birmingham vasculitis activity score; ESR: erythrocyte sedimentation rate; CRP: C-reactive protein; NFI: novel fibrosis index.
